# Relationship between maternal caffeine and coffee intake and pregnancy loss: A grading of recommendations assessment, development, and evaluation-assessed, dose-response meta-analysis of observational studies

**DOI:** 10.3389/fnut.2022.886224

**Published:** 2022-08-09

**Authors:** Alireza Jafari, Sina Naghshi, Hossein Shahinfar, Sayed Omid Salehi, Fateme Kiany, Mohammadreza Askari, Pamela J. Surkan, Leila Azadbakht

**Affiliations:** ^1^Department of Community Nutrition, School of Nutritional Sciences and Dietetics, Tehran University of Medical Sciences, Tehran, Iran; ^2^Physiology Research Center, Institute of Neuropharmacology, Kerman University of Medical Sciences, Kerman, Iran; ^3^Department of Clinical Nutrition, School of Nutritional Sciences and Dietetics, Tehran University of Medical Sciences, Tehran, Iran; ^4^Department of Nutrition, Iran University of Medical Sciences, Tehran, Iran; ^5^Student Research Committee, Faculty of Public Health, Iran University of Medical Sciences, Tehran, Iran; ^6^Department of Nutrition, School of Allied Medical Sciences, Ahvaz Jundishapur University of Medical Science, Ahvaz, Iran; ^7^Department of International Health, Johns Hopkins Bloomberg School of Public Health, Baltimore, MD, United States; ^8^Diabetes Research Center, Endocrinology and Metabolism Clinical Sciences Institute, Tehran University of Medical Sciences, Tehran, Iran; ^9^Department of Community Nutrition, School of Nutrition and Food Science, Isfahan University of Medical Science, Isfahan, Iran

**Keywords:** caffeine, coffee, risk, meta-analysis, pregnancy loss

## Abstract

**Background:**

Numerous studies report an association between coffee or caffeine consumption and pregnancy loss; however, the nature and strength of this relationship have not been clearly established. Based on recent studies, our meta-analysis aimed to test whether a dose–response relationship between coffee or caffeine consumption and pregnancy loss exists.

**Methods:**

We searched for articles in PubMed, Web of Science, and Scopus published until May 2022. Two independent reviewers extracted data and rated the quality of the evidence using the GRADE approach. We applied a random-effects, one-stage dose–response meta-analysis.

**Results:**

A total of 34 articles (18 cohort studies and 16 case-control studies) were included in this review. Results showed a significantly higher risk of pregnancy loss for coffee consumption before (Pooled ES: 1.21; 95% CI: 1.01–1.43) and during pregnancy (Pooled ES: 1.26; 95% CI: 1.04–1.57), and for coffee consumption during pregnancy in case-control studies (Pooled ES: 1.20; 95% CI: 1.19–6.41). Findings from this meta-analysis demonstrated that caffeine intake during pregnancy was associated with a significantly higher risk of pregnancy loss in cohort (Pooled ES: 1.58; 95% CI: 1.23–2.01) and case-control studies (Pooled ES: 2.39; 95% CI: 1.69–3.37, *P* < 0.001), respectively. A dose–response analysis suggested that an increase of a cup of coffee per day during pregnancy was associated with 3% increased risk of pregnancy loss; 100 mg of caffeine per day during pregnancy was also associated with 14 and 26% increased risk of pregnancy loss in cohort and case-control studies, respectively. A non-linear dose–response association was observed between coffee intake and the risk of pregnancy loss.

**Conclusion:**

This study confirms that coffee or caffeine consumption raises the risk of pregnancy loss. Researchers are encouraged to conduct more studies to explore the underlying mechanisms and active compounds in coffee and caffeine.

**Systematic Review Registration:**

[www.crd.york.ac.uk/prospero/], identifier [CRD42021267731].

## Introduction

Fetal deaths account for a high percentage of perinatal deaths and can be categorized into stillbirth and spontaneous abortion (or miscarriage) (1, 2). Miscarriage or spontaneous abortion is defined as the involuntary termination of a pregnancy leading to fetal death before week 20 of gestation. Stillbirth refers to the death of a fetus after 20 weeks of pregnancy or after attaining 14 oz in weight. In other situations, the fetus is alive at the beginning of labor but dies during delivery (3). An estimated 26% of all pregnancies and up to 10% of clinically recognized pregnancies result in pregnancy loss (4), whereas the global stillbirth rate was 18.4 per 1,000 total births in 2015 (5). Risk factors for fetal death include advanced maternal age, obesity/overweight, low socioeconomic status, history of fetal loss, smoking and alcohol consumption, and caffeine intake during pregnancy (6).

Caffeine is a 1,3,7-trimethylxanthine; in the world, it is one of the most common substances consumed in pharmacologically active amounts and is found in beverages like coffee, tea, soda, solid milk chocolate, and products containing cacao (7, 8). Hoyt et al. reported that 82% of pregnant women in the United States consumed caffeine (9). In another study, the National Institute of Child Health and Human Development (NICHD) reported that 35% of participants consumed coffee and 41% drank soda daily (10). A safe dose of daily caffeine intake in pregnancy is not more than 200 mg (or two cups) of coffee (11). In adults, caffeine is metabolized in the liver mainly by cytochrome P450 enzymes (monooxygenase and xanthine oxidase enzymes). However, since the P450 enzyme system remains undeveloped until infancy, there is a low metabolite rate in the fetus (12). Caffeine has delayed clearance in the second and third trimesters of pregnancy compared with clearance in non-pregnant women. Ingested caffeine by pregnant women is rapidly absorbed from the digestive tract and readily passes through the placenta (13, 14). In pregnant women, caffeine has a long clearance compared with non-pregnant women, and the fetus has a low metabolite rate because of the lack of enzymes required in caffeine metabolism (6, 15, 16). The half-life of caffeine is tripled in the second and third trimesters of pregnancy compared to in non-pregnant women, and therefore the fetus is more exposed to caffeine and its metabolites (6, 13). Caffeine raises cellular cyclic adenosine monophosphate levels, which can accelerate cell growth; it also raises levels of circulating catecholamine that could interfere with placental blood flow through vasoconstriction and lead to fetal hypoxia (13, 17, 18). During pregnancy, caffeine consumption has been connected to different adverse outcomes, including spontaneous abortion, congenital disabilities, and low birth weight (19). Although some observational studies have been carried out to investigate how caffeine intake is associated with the risk of fetal loss, the results have not been consistent due to difficulties in measuring self-reported caffeine intakes, different caffeine metabolisms, and differences in study settings and genetics of participants (8, 16, 20).

The previous meta-analysis including studies published until 2015 (2, 21, 22) was limited in scope due to inclusion restrictions. We had fewer limitations on inclusion criteria, and we also included more recent studies (8, 16, 20, 23–27). Moreover, we use a new one-stage random effect dose–response analytic approach. Therefore, in the present meta-analysis, we aimed to update and expand the current literature on the association of caffeine and coffee consumption with the risk of miscarriage.

## Methods

This systematic review and meta-analysis were performed based on PRISMA (Preferred Reporting Items for Systematic Reviews and Meta-Analyses) and MOOSE (Meta-analyses Of Observational Studies in Epidemiology) guidelines (28, 29). The corresponding checklists are shown in Supplementary Tables 1, 2.

### Literature search

A systematic literature search was conducted on Medline/PubMed, ISI Web of Knowledge, and Scopus to include observational studies published until May 2022. The following keywords were used in a comprehensive literature search: [“caffeine*” OR “coffee*”] in combination with [“miscarriage*,” “abortion*,” “stillbirth*,” “still birth*,” “fetal loss*,” “fetal death*,” “misbirth*,” and “pregnancy loss*”] AND [“prospective*” OR “retrospective*” OR “observational” OR “longitudinal” OR “cohort*” OR “relative risk” OR “hazard ratio” OR “odds ratio” OR “follow-up” OR “follow up” OR “population-based” OR “hr” OR “rr”]. The reference lists of full-text publications were also screened to identify any relevant studies. The search was not restricted by publication date or language. Supplementary Table 3 provides more details on the search terms.

### Study selection

Endnote (version 20) was used for the management of records downloaded from databases. Two reviewers (AJ and MA) screened the titles and abstracts of each relevant study. Potentially eligible studies were reviewed independently by the reviewers. Discrepancies were resolved by discussion, and if necessary, a third author (LA) was consulted to reach consensus.

The two researchers independently checked the studies’ eligibility based on our inclusion and exclusion criteria, and differences were resolved by arbitration or consensus. Original studies were selected if they met the following inclusion criteria:

1. Study design: cohort or case-control studies.
2. Participants: pregnant women.
3. Exposure: dietary caffeine or coffee intake.
4. Outcome: pregnancy loss including miscarriage, abortion, and stillbirth (RR, OR, HR with 95% confidence interval [CI]).

### Data extraction

Two independent reviewers (AJ and SN) extracted the relevant data independently. Any disagreements and differences were resolved by the study supervisor (LA). The following data were extracted from each included study by use of a standardized data-collection form: the first author’s last name, publication year, country, study design, length of follow-up, sample size, the incident of pregnancy loss, measurement of exposure, method of dietary intake assessment and outcome, comparison categories, and effect sizes (RR, OR, HR) with 95% CI with the maximum number of adjustments. Disagreements were resolved through discussion with the senior author (LA).

### Risk of bias assessment

The risk of bias in the included studies was assessed by two authors using the ROBINS-I tool (30). This tool consists of seven questions aimed at determining bias based on confounding, participant selection, exposure classification, bias due to departures from intended exposures, missing data, outcomes measurement, and selection of the reported result. Discrepancies were resolved through discussion with the senior author. We used the Grading of Recommendations Assessment, Development, and Evaluation (GRADE) approach to evaluate the quality of the evidence (31).

### Statistical analysis

In this meta-analysis, the common measure of association was OR in case-control studies and RR in cohort studies. Due to the low incidence of pregnancy loss, ORs and RRs are considered nearly equivalent in cohort studies (32). If an estimate was reported for the lowest category of caffeine or coffee intake compared with the highest category, we computed the highest vs. lowest estimates using the Orsini method (33). Results from case-control and cohort studies were presented separately. The Q-statistic and *I*^2^ were used as indicators of heterogeneity. We used a random-effects model (*n* > 5) to assess heterogeneity (34) for more conservative results than a fixed-effects model (35). We conducted a series of subgroup analyses to identify potential sources of heterogeneity based on the study design while controlling for BMI, alcohol consumption, smoking, education, and vitamin supplementation. We also performed an analysis that, excluded or included studies one-by-one to assess the influence overall estimate by a single study. When at least 10 primary studies had available data, we used Egger’s regression asymmetry test (36) and/or used a visual examination of counter-enhanced funnel plots (37) to detect the effects of potential publication bias from these studies (38).

Greenland and Orsini’s (39, 40) method was used to compute the trend based on the odds ratios, relative risks, or hazard ratios estimates and their respective 95% confidence intervals across categories of 100 mg/day increments of caffeine and intake of one cup of coffee per day. This method requires the distribution of cases and the odds ratios, relative risks, or hazard ratios with the variance estimates for three or more quantitative categories of exposure. We used mean or median intake, midpoint, and estimated the midpoint (if the mean was not presented) to derive the dose–response trend.

We applied a one-stage weighted, random-effects dose–response meta-analysis to investigate possible associations between caffeine or coffee intake and pregnancy loss. This non-linear dose–response analysis was done by modeling caffeine intake with a restricted cubic spline with 3 knots fixed at 10, 50, and 90% (41) using a generalized least squares trend estimation method. Furthermore, we combined the study-specific estimates by the restricted maximum likelihood method into a random-effects model. A probability value for non-linearity was estimated by testing the null hypothesis, in which the coefficient of the second spline was considered equal to zero (33). Statistical analyses were conducted using STATA version 16.0 (StataCorp, College Station, TX, United States), and meta and dr meta were used for analysis. A two-sided *P*-value < 0.05 was considered significant for all tests, including Cochran’s *Q*-test.

## Results

### Literature search

**FIGURE 1:**
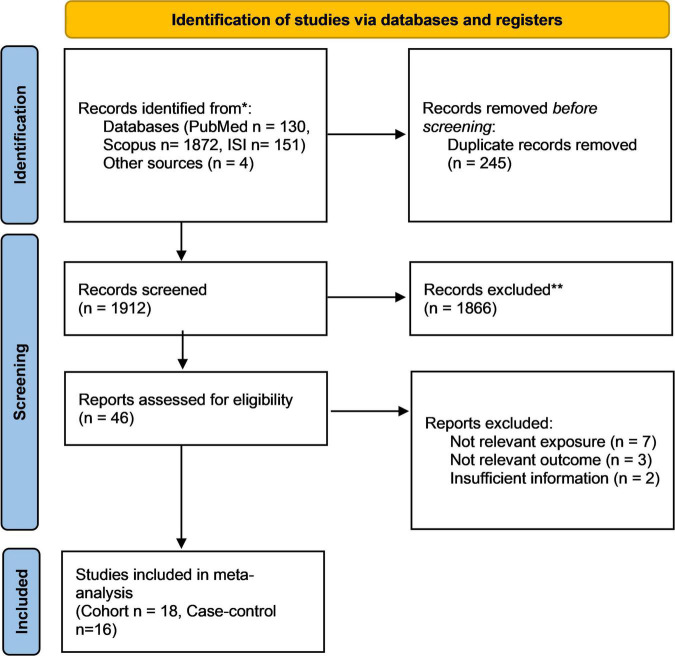
Flowchart of study selection.

We identified 2,253 records in our initial search of three databases. We removed 245 duplicates and 1,912 non-relevant articles *via* title and abstract screening. After excluding those that did not meet the inclusion criteria, we identified 46 full-text publications of potentially relevant studies. After a full-text review, we excluded an additional nine studies (42–50). The flowchart is shown in Figure 1.

Finally, 18 cohort studies and 16 case-control studies were included in the current systematic and meta-analysis: seven studies reported effect sizes for coffee consumption, 25 for caffeine intake, and 2 studies reported both. Of these publications, 6 reported effect sizes for coffee consumption before pregnancy, 10 reported effect sizes for caffeine consumption before pregnancy, 12 reported effect sizes for coffee consumption during pregnancy, and 24 reported effect sizes for caffeine intake during pregnancy.

### Characteristics of included studies

Characteristics of the studies are provided in Tables 1, 2. Participants in these studies ranged from 66 to 90,086 people. In total, 292,795 participants were included in the 34 publications in the systematic review. In total, 12 (31,544 participants) publications were conducted in the United States and 22 (261,251 participants) in non- United States countries.

**TABLE 1:** Characteristic of included cohort studies.

References	Country	Design	Cases	Participants	Incidence	Exposure	Assessment	Period	Outcome	Adjustments
Axelsson et al. (23)	Sweden	Cohort	126	1242	10.1	Coffee	Self-administered questionnaire	Month before pregnancy, First trimester	Miscarriage	Age, year, occupation, smoking, illness, medication
Wilcox et al. (54)	United States	Cohort	43	171	25	Caffeine	Interview	Pregnancy	Pregnancy loss	
Armstrong et al. (53)	Canada	Cohort	7760	35848	21.6	Coffee	Interview	First trimester	Spontaneous abortion	Maternal age, educational, ethnic, employment
Mills et al. (19)	United States	Cohort	59	423	13.9	Caffeine	Interview	First trimester	Spontaneous abortion	Smoking, age, parity, prior SAB, alcohol, education, income
Fenster et al. (7)	United States	Cohort	498	5144	9.6	Caffeine	Interview	Before pregnancy, First trimester	Spontaneous abortion	Age, smoking, alcohol, pregnancy history, race, employment, marital, socioeconomic
Dominguez-Rojas et al. (56)	Spain	Cohort	169	691	24	Caffeine	Interview	Pregnancy	Spontaneous abortion	Age, marital, previous SAB
Dlugosz et al. (57)	United States	Cohort	135	2967	4.6	Caffeine	Interview	First month	Spontaneous abortion	Age, gestational stage
Wisborg et al. (17)	Denmark	Cohort	82	18478	0.4	Coffee	Self-administered questionnaire	First trimester	Stillbirth	Smoking, alcohol, parity, age, marital, education, employment, BMI
Khoury et al. (25)	United States	Cohort	103	191	53	Caffeine	Interview	First trimester	Spontaneous abortion	Age, years since diagnosis of diabetes, previous SAB, nephropathy, retinopathy, glycemic control, smoking
Bech et al. (13)	Denmark	Cohort	1102	88482	1.2	Coffee	Interview	First to early second trimester	Stillbirth	Age, parity, smoking, BMI, alcohol, socio-occupational
Savitz et al. (18)	United States	Cohort	258	2407	10.7	Caffeine	Interview	Pre-pregnancy	Miscarriage	age, race/ethnicity, education, marital, alcohol, vitamin, nausea, vomiting
Weng et al. (15)	United States	Cohort	172	1063	16.2	Caffeine	Interview	Early pregnancy	Miscarriage	Age, race, education, income, marital, previous miscarriage, nausea vomiting, smoking, alcohol, Jacuzzi, magnetic fields.
Pollack et al. (66)	United States	Cohort	13	66	19.6	Caffeine	Self-administered questionnaire	Sensitive windows	Miscarriage	Age, alcohol, smoking, prior SAB
Greenwood et al. (65)	United Kingdom	Cohort	28	2635	1.1	Coffee, Caffeine	Self-administered questionnaire	First trimester	Late spontaneous abortion, stillbirth	Age, parity, smoking, alcohol
Hahn et al. (16)	Denmark	Cohort	732	5132	14.3	Caffeine	Self-administered questionnaire	Pregnancy	Spontaneous abortion	Age, physical activity, parity, BMI, vocational training/education, smoking, prior SAB
Morales-Suárez-Varela et al. (8)	Denmark	Cohort	1178	90086	1.3	Caffeine	Interview	Pregnancy	Fetal death	Age, parity, socio-economic, physical exercise, alcohol, BMI
Gaskins et al. (20)	United States	Cohort	2756	15950	17.2	Caffeine	FFQ	Pregnancy	Spontaneous abortion	Age, year, energy, BMI, smoking, physical activity, history of infertility, marital, employment, shift work, race, alcohol, supplemental folate
Purdue-Smithe et al. (26)	United States	Cohort		1228		Caffeine	Self-administered questionnaire	Pregnancy	Pregnancy loss	Nausea and vomiting

**TABLE 2:** Characteristic of included case-control studies.

References	Country	Design	Cases	Participants	Incidence	Exposure	Assessment	Period	Outcome	Adjustments
Parazzini et al. (24)	Italy	Case-control	78	212	3.6	Coffee	Interview	First trimester	Miscarriage	Age
Fenster et al. (69)	United States	Case-control	607	1284	3.2	Caffeine	Interview	First trimester	Spontaneous abortion	Age, race, marital status, insurance coverage, smoking, alcohol, previous SAB
Infante-Rivard et al. (64)	Canada	Case-control	331	1324	25	Caffeine	Interview	Pregnancy	Fetal loss	Period of pregnancy, age, education, smoking, alcohol, uterine abnormality, work schedule
Al-Ansary et al. (55)	Saudi Arabia	Case-control	226	226		Caffeine	Interview	Pregnancy	Spontaneous abortion	
Parazzini et al. (70)	Italy	Case-control	782	1543	50.6	Coffee	Interview	Before pregnancy, First trimester	Spontaneous abortion	Age, education, previous live births and miscarriages, maternal alcohol drinking and smoking in the first trimester of pregnancy and nausea intensity
Cnattingius et al. (58)	Sweden	Case-control	562	1515	37	Caffeine	Interview	Pregnancy	Spontaneous abortion	Age; number of previous pregnancies; history of SAB; alcohol, nausea, vomiting, fatigue
Wen et al. (59)	United States	Case-control	75	650	11.5	Caffeine	FFQ	Before pregnancy, First trimester	Spontaneous abortion	
Tolstrup et al. (52)	Denmark	Case-control	303	1684	18	Caffeine	FFQ	Pre-pregnancy	Spontaneous abortion	Age, marital, smoking, alcohol
Giannelli et al. (60)	United Kingdom	Case-control	160	474	33.7	Coffee, Caffeine	Interview	Before and during pregnancy	Miscarriage	Age, nausea
Rasch et al. (51)	Denmark	Case-control	330	1498	22	Caffeine	structured questionnaire	Pregnancy	Spontaneous abortion	Age, parity, occupation, cigarette, alcohol
Matijasevich et al. (6)	Uruguay	Case-control	382	1174	32.5	Caffeine	Interview	Pregnancy	Fetal death	education, history of SAB, vomiting/nausea, attendance for prenatal care
Maconochie et al. (61)	United Kingdom	Case-control	603	6719	8.9	Caffeine	Questionnaire	Pre-pregnancy	Miscarriage	Age, history, nausea
Agnesi et al. (62)	Italy	Case-control	123	231	0.53	Coffee	Interview	Pregnancy	Spontaneous abortion	Age, education
Zhang et al. (71)	China	Case-control	326	726	44.9	Caffeine	structured questionnaire	Pregnancy	Spontaneous abortion	Age, education, BMI, history, smoking, alcohol
Stefanidou et al. (63)	Italy	Case-control	52	312	16.6	Caffeine	Interview	Pregnancy	Sine causa recurrent miscarriage	Age, tobacco, education
Heazell et al. (27)	United Kingdom	Case-control	290	1019	28.4	Caffeine	Interview	Pregnancy	Late stillbirth	Ethnicity, age, BMI, smoking, education, parity, gestation, birth weight centile, dietary supplements

To examine coffee and caffeine intake, 26 studies used dietary records or recall, and 8 studies used a food frequency questionnaire.

Most cohorts controlled for some conventional risk factors, including age (*n* = 30), body mass index (*n* = 7), smoking (*n* = 17), education (*n* = 13), alcohol consumption (*n* = 17), and previous spontaneous abortion (SAB; *n* = 12). Some others also adjusted for energy intake (*n* = 1) and other dietary variables (*n* = 1).

Supplementary Figure 1 displays the results of the risk of bias assessment. Most of the review studies had a serious risk of bias, and only seven studies (8, 15, 20, 24, 51–53) showed a moderate risk of bias. Regarding confounding, bias was considered serious (6, 24, 54–63) or moderate. Selection bias was considered moderate in two studies (19, 61) and serious in one study (25). One study (19) had low bias in the misclassification domain, since bias in the remaining studies was moderate. Missing data were measured in four studies (15, 27, 59, 61). Measurement bias was determined to be low and moderate (13, 19, 24, 52, 57, 64–66). As for reporting bias, only one article (54) showed a moderate risk of bias.

The GRADE assessment was very low for caffeine intake during pregnancy, low for coffee and caffeine intake before pregnancy, and moderate for coffee intake during pregnancy (Supplementary Table 4).

### Findings from the meta-analysis on coffee intake

**FIGURE 2:**
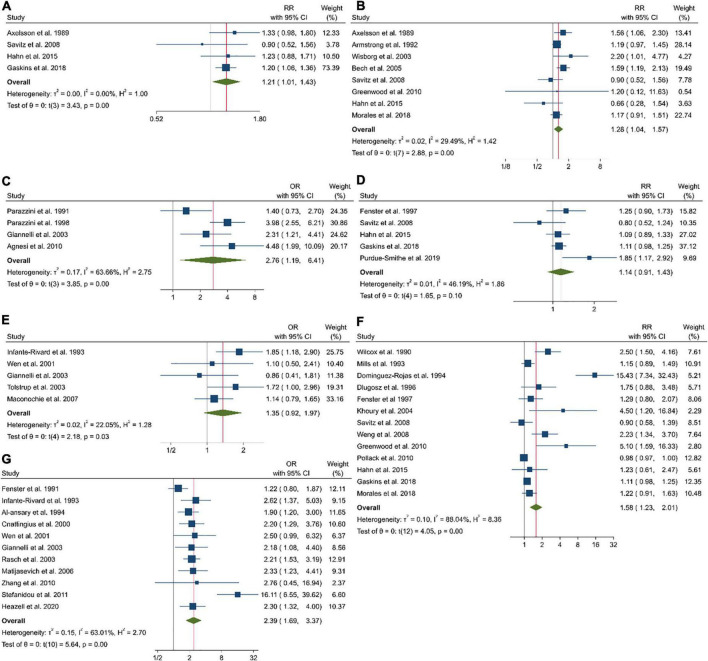
Forest plots of the estimated relative risk (RR) of pregnancy loss related to coffee intake before pregnancy in cohort studies **(A)**, coffee intake during pregnancy in cohort studies **(B)**, coffee intake during pregnancy in case-control studies **(C)**, caffeine intake before pregnancy in cohort studies **(D)**, caffeine intake before pregnancy in case-control studies **(E)**, caffeine intake during pregnancy in cohort studies **(F)**, and caffeine intake during pregnancy in case-control studies **(G)**.

Coffee consumption before pregnancy, which was examined in four cohort and two case-control studies with a total of 26,748 participants and 4,817 pregnancy losses, was associated with an increased risk of pregnancy loss (Pooled ES comparing the highest and lowest intakes: 1.21; 95% CI: 1.01–1.43, *P* < 0.001), with no significant heterogeneity among the studies (τ^2^ = 0, *I*^2^ = 0%; *P*_heterogenity_ = 0.68; Figure 2A).

Examining the association between coffee consumption and the risk of pregnancy loss in eight cohorts and four case-control studies that involved a total of 246,770 participants and 12,409 abortions, we found a significantly higher risk (Pooled ES comparing the highest and lowest intakes: 1.28; 95% CI: 1.04–1.57, *P* < 0.001), with no significant heterogeneity among the cohort studies (τ^2^ = 0.02, *I*^2^ = 24.49%; *P*_heterogenity_ < 0.001; Figure 2B) and case-control studies (2.76; 95% CI: 1.19–6.41; τ^2^ = 0.17, *I*^2^ = 63.66%; Figure 2C).

### Findings from the meta-analysis on caffeine intake

The association between caffeine intake before pregnancy and the risk of pregnancy loss was examined in five cohort and five case-control studies that included 40,712 participants with 5,716 cases, we found a non-significant association (Pooled ES comparing the highest and lowest intakes: 1.14; 95% CI: 0.91–1.43, *P* = 0.02), with moderate heterogeneity among the cohort studies (τ^2^ = 0.01, *I*^2^ = 46.19%; *P*_heterogenity_ = 0.1; Figure 2D) and case-control studies (1.35; 95% CI: 0.92–1.97, *P* = 0.03; τ^2^ = 0.02, *I*^2^ = 22.05; Figure 2E).

A total of 13 cohort and 11 case-control studies examined associations between caffeine intake during pregnancy and the risk of pregnancy loss. These studies included a total of 137,128 participants among them, 9,666 cases of pregnancy loss were found. The summary effect size for abortion, comparing highest and lowest caffeine intake, was 1.58 (95% CI: 1.23–2.01, *P* < 0.001), indicated a significant positive association between caffeine intake and the risk of pregnancy loss (Figure 2F). High heterogeneity among the studies was observed (τ^2^ = 0.10, *I*^2^ = 88.04%; *P*_heterogenity_ < 0.001). Notably, 11 studies reported on the association in case-control studies (2.39; 95% CI: 1.69–3.37; τ^2^ = 0.15, *I*^2^ = 63.01; Figure 2G).

### Linear and non-linear dose–response analysis

**FIGURE 3:**
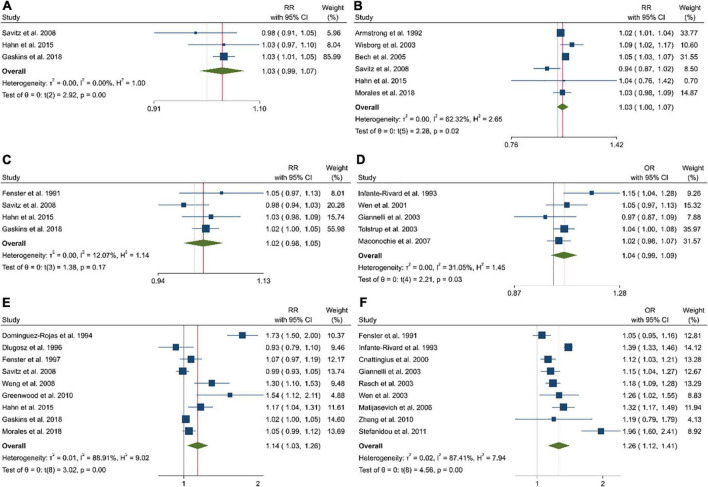
The linear dose–response analysis between the risk of pregnancy loss and one cup of coffee intake before pregnancy in cohort studies **(A)**, one cup of coffee intake during pregnancy in cohort studies **(B)**, 100 mg caffeine intake before pregnancy in cohort studies **(C)**, 100 mg caffeine intake before pregnancy in case-control studies **(D)**, 100 mg caffeine intake during pregnancy in cohort studies **(E)**, and 100 mg caffeine intake during pregnancy in case-control studies **(F)**.

In the dose–response analysis of coffee intake before pregnancy and abortion risk based on four studies, we found a significant positive non-linear association (*P*_non–linearity_ = 0.976; Figure 3A). A linear dose–response analysis revealed a significantly higher risk of abortion with each additional cup per day (Pooled ES: 1.03; 95% CI: 0.99–1.07, *P* = 0.002; Figure 2A).

Eight of 10 studies that had sufficient data to examine the association between coffee intake during pregnancy and the risk of pregnancy loss were included in the dose–response analysis (Figure 3B). Coffee intake was associated with a higher risk of pregnancy loss (*P*_non–linearity_ = 0.036). This was also the case in the linear dose–response meta-analysis when examining an additional one cup of coffee per day (Pooled ES: 1.03; 95% CI: 1.00–1.07, *P* = 0.019; Figure 2B).

A non-linear dose–response analysis of eight studies revealed a significant positive association between caffeine intake before pregnancy and abortion (*P*_non–linearity_ = 0.929; Figure 3C). Based on the linear dose–response analysis, an additional 100 mg of caffeine per day was associated with a higher risk of abortion in the cohort (Pooled ES: 1.02; 95% CI: 0.98–1.05, *P* = 0.11; Figure 2C) and case-control studies (Pooled ES: 1.04; 95% CI: 0.99–1.09; Figure 2D).

**FIGURE 4:**
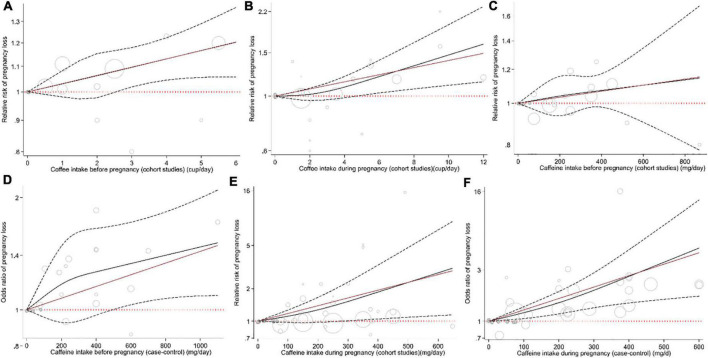
Non-linear dose–response association between the risk of pregnancy loss and intake of coffee before pregnancy in cohort studies **(A)**, intake of coffee during pregnancy in cohort studies **(B)**, intake of caffeine before pregnancy in cohort studies **(C)**, intake of caffeine before pregnancy in case-control studies **(D)**, intake of caffeine during pregnancy in cohort studies **(E)**, and intake of caffeine during pregnancy in case-control studies **(F)**.

Combining data from 16 (out of 20 studies) in the dose–response analysis of caffeine intake during pregnancy and abortion risk, a significant non-linear association was observed in both cohort (*P*_non–linearity_ = 0.085; Figure 4E) and case-control studies (*P*_non–linearity_ = 0.372; Figure 4F). Moreover, the linear association between an increase of 100 mg of caffeine per day was associated with 14% and 26% increased risk of pregnancy loss in the cohort (Pooled ES: 1.14; 95% CI: 1.03–1.26, *P* < 0.001; Figure 2E) and case-control studies (Pooled ES: 1.26; 95% CI: 1.12–1.41; Figure 2F). No other association was observed (Figures 3D–F, 4A–D).

### Subgroup, sensitivity analyses, and publication bias

To test the robustness of the findings, we conducted subgroup analyses to find possible sources of heterogeneity. These analyses were performed based by controlling for history of pregnancy loss, BMI, alcohol consumption, smoking, education, marital status, employment, nausea, race, and vitamin intake. Supplementary Figures 2–8 present findings for the different subgroups. A non-significant association was seen between coffee consumption before pregnancy and the risk of pregnancy loss in studies that controlled for education but did not control for the history of pregnancy loss (Supplementary Figure 2). A significant positive association was seen between coffee consumption during pregnancy and the risk of pregnancy loss among studies that controlled for pregnancy loss history, smoking, employment, and studies that did not control for BMI, education, nausea, and vitamin intake (Supplementary Figure 3). In terms of coffee intake during pregnancy and the risk of pregnancy loss, a significant positive risk was seen in case-control studies that did not control for education and nausea (Supplementary Figure 4). A non-significant association was observed between caffeine consumption before pregnancy and the risk of pregnancy loss in cohort studies that controlled for alcohol, marital status, BMI, education, nausea, race, and vitamin intake and did not control for pregnancy loss history, smoking, alcohol, marital status, employment, and race (Supplementary Figure 5). A significant positive association was seen between caffeine consumption before pregnancy and the risk of pregnancy loss in case-control studies among those that controlled for smoking, alcohol, employment, education, and studies that did not control for marital status and nausea (Supplementary Figure 6). In terms of caffeine intake during pregnancy and the risk of pregnancy loss in cohort studies, a non-significant positive association was seen in case-control studies that did control for education, nausea, and vitamin intake (Supplementary Figure 7). A non-significant association was seen between caffeine consumption during pregnancy and the risk of pregnancy loss in case-control studies that controlled for marital status (Supplementary Figure 8).

Regarding the significant positive association between caffeine intake and the risk of pregnancy loss, findings from the sensitivity analyses indicated that this association was dependent on particular studies. For example, exclusion of studies by Gaskins et al. (Supplementary Figure 9a), Wen et al., Giannelli et al., and Maconochie et al. (Supplementary Figure 9e) resulted in a non-significant association between caffeine intake and pregnancy loss. When we excluded the study by Savitz et al., pooled effect estimates resulted in a significant association (Supplementary Figure 9d).

Findings from another sensitivity analysis revealed that excluding any single study from the analysis did not appreciably alter the pooled effect sizes (Supplementary Figures 9b,c,f,g). No publication bias was found based on Egger’s regression asymmetry test (Supplementary Figures 10, 11). In terms of caffeine intake during pregnancy and pregnancy loss, Egger’s linear regression test indicated some degree of publication bias; however, the trim and fill methods’ application did not change the average effect size, further suggesting that results were not affected by publication bias. Three missing studies were imputed in regions of the contour-enhanced funnel plots to adjust for asymmetry (Supplementary Figure 9a).

## Discussion

### Summary of the findings

In summary, these meta-analysis results show pregnancy loss was associated with coffee intake before and during pregnancy, with caffeine during pregnancy, but not with caffeine intake before pregnancy. Based on a linear dose–response analysis, increased intake of one cup of coffee during pregnancy was associated with 3% increased risk of pregnancy loss. Likewise, increased intake of 100 mg of caffeine per day during pregnancy increased the risk of pregnancy loss by 14 and 26% based on cohort studies and case-control studies, respectively.

### Comparisons with other studies

In this study, we found that higher coffee intake before pregnancy was associated with an increased risk of pregnancy loss, which is consistent with the cohort study by Gaskins et al. that showed that higher coffee consumption before pregnancy could increase the risk of miscarriage (RR = 1.21; 95% CI: 1.07–1.37, *P* = 0.002) and that consuming ≥4 servings/day had 20% increased risk of SAB (20). However, Savitz et al. observed a null relationship between coffee consumption before pregnancy and the risk of miscarriage (18).

Our findings revealed that increased coffee consumption during pregnancy was related to an increase in the risk of pregnancy loss in both cohort and case-control studies (8). However, Morales et al.’s cohort study found no significant association (8). Our results are consistent with the meta-analysis study by Li et al., who discovered a significant positive association between coffee intake and the risk of pregnancy loss (RR = 1.31; 95% CI: 1.15–1.50, *P* < 0.001) but not for low (<2 cups) and moderate (2–3 cup) consumption in subgroup analyses (22). Likewise, in both meta-analyses, no significant heterogeneity was observed. On the contrary, Savitz et al.’s cohort study showed no association between coffee consumption during pregnancy and pregnancy outcomes (18). Although the harmful effects of coffee on pregnancy loss appear to be due to caffeine, it should be noted that in the case of low coffee intake, other coffee compounds such as amino acids, carbohydrates, vitamins, and minerals can reduce caffeine’s harmful effects (8). Our results showed that an increment of one cup of coffee was correlated with 3% increased risk of pregnancy loss. Similar to our results, Li et al. revealed that every increase of two cups of coffee was associated with a 3% increase in the risk of pregnancy loss (22).

With regard to caffeine, we found no significant association between its intake before pregnancy and the risk of pregnancy loss in either cohort or case-control studies. In line with our study, in the studies by Gaskins et al. and Tolstrup et al., caffeine consumption before pregnancy increased the risk of miscarriage (20, 52). Regarding the dose–response analysis, we found no significant association between caffeine intake and the risk of pregnancy loss. Consistent with our study, Gaskins et al.’s study showed no evidence of a non-linear association (*P* = 0.06) (20). Our inclusion of both cohort and case-control studies, more recent studies (than in prior reviews), and no evidence of publication bias may have led to the differences between studies.

We found a positive association between caffeine consumption during pregnancy and the risk of pregnancy loss. Our findings are in agreement with several past studies (2, 16, 21). Li et al. found that caffeine consumption was associated with the risk of pregnancy loss (22). Both meta-analyses by Greenwood et al. and Li et al. included cohort and case-control studies (21, 22). On the contrary, we performed subgroup and sensitivity analysis to reduce heterogeneity. Additionally, 100 mg of additional caffeine per day during pregnancy was associated with an increased risk of pregnancy loss by 14%, which is in agreement with the findings of Greenwood et al. On the contrary, Greenwood et al. observed a non-linear relationship between caffeine consumption and abortion risk (21). Conversely, caffeine intake during pregnancy was not related to abortion risk in two cohort studies (18, 66); this may be because of the small sample sizes and/or because of including only miscarriage.

Smoking is related to caffeine intake and is a known risk factor for pregnancy loss. Therefore, smoking is a potentially important confounder of the association between caffeine intake and pregnancy loss. Pregnancy symptoms (including nausea, vomiting, and aversions to smells and tastes) are more common in healthy pregnancies than in pregnancies that end in spontaneous abortion. Women with viable pregnancies may be more likely to reduce their caffeine consumption in response to these symptoms during pregnancy. Symptoms during pregnancy can affect the interpretation of the relationship between caffeine and pregnancy loss.

### Possible biological mechanisms

As mentioned, the main component of coffee is caffeine, which has several effects on the human body, especially pronounced during the pregnancy period. Caffeine can increase catecholamine secretion and reduce uterine and placental blood flow due to its vasoconstrictive effects, resulting in fetal hypoxia and growth and developmental defects. Moreover, caffeine can directly affect the fetal cardiovascular function and initiate tachycardia (2). However, some studies did not emphasize the harm of caffeine consumption during pregnancy, especially for pregnancy loss (18). Despite such contradictory findings, caffeine restriction may help inform this debate; however, in a systematic review of RCTs, Jahanfar et al. did not conclude that caffeine restriction had an effect on pregnancy outcomes (67). Caffeine consumption can reduce nausea and discomfort in pregnancy by lowering estrogen levels in the blood, but lowering blood estrogen levels can increase the risk of miscarriage. However, several factors can affect the relationship between caffeine consumption and the risk of pregnancy loss, nausea severity, and the amount of caffeine consumption for reducing nausea (59). Another important confounder between caffeine consumption and the risk of pregnancy loss is smoking, because smoking is associated with coffee consumption (2). In addition, other factors like circulating caffeine levels and their metabolites, genetic differences in caffeine metabolism, and different lifestyles may confound the relationship between caffeine consumption and the risk of pregnancy loss (67).

### Strengths and limitations

Our study is a comprehensive and up-to-date meta-analysis that investigated the association of maternal coffee and caffeine with the risk of pregnancy loss. Also, we used the one-stage weighted effect method. There are several strengths of our study. We tried to reduce the effects of confounding, searched databases without language restrictions, and included a large number of studies by modulating the effects of confounders in the meta-analysis. Additionally, we evaluated coffee and caffeine consumption separately and evaluated time points during and before pregnancy. Moreover, we conducted a one-stage dose–response analysis to determine linear and non-linear relationships between the variables, performed a subgroup analysis to eliminate the possible effects of confounders (such as BMI, alcohol consumption, smoking, education, and taking vitamins), and performed Egger’s asymmetry test to assess the effect of publication bias.

The limitations of this study include potential measurement error of caffeine intake in the primary studies, the use of case-control studies, the possibility of recall and selection bias, and evaluation of coffee consumption as a source of caffeine. Regarding the measurement error of caffeine intake, the studies examined in our meta-analysis used interviews and self-report as well as a food frequency questionnaire (FFQ), which are accurate and reliable (68).

## Conclusion

This meta-analysis suggests that increased coffee and caffeine consumption in pregnancy may be associated with an increased risk of pregnancy loss. More research is needed to explore the underlying mechanisms and active compounds in coffee and caffeine.

## Data availability statement

The raw data supporting the conclusions of this article will be made available by the authors, without undue reservation.

## Author contributions

AJ and MA designed the study and independently carried out the literature search and screening of manuscripts. SN analyzed the data. AJ and HS assessed risk of bias and quality of evidence. AJ, SN, SS, and FK wrote the manuscript. PS edited the English and commented on the manuscript. LA supervised and revised the study. All authors have read and approved the final version of the manuscript.
