# Supplementary material for: Relationship between maternal caffeine and coffee intake and pregnancy loss: A grading of recommendations assessment, development, and evaluation-assessed, dose-response meta-analysis of observational studies
Source: Front Nutr. 2022 Aug 9;9:886224. doi: 10.3389/fnut.2022.886224 (PMC9396037; doi:10.3389/fnut.2022.886224)
Supplement: Supplementary file 1 [file Data_Sheet_1.pdf]

Online Supplemental Material

Relationship between maternal caffeine and coffee intake and pregnancy loss: a GRADE-assessed, dose-response meta-analysis of observational studies

Alireza Jafari[1,2], Sina Naghshi[3], Hossein Shahinfar[4,5], Sayed Omid Salehi[3], Fateme Kiany[6], Mohammadreza Askari[2], Pamela J Surkan[7], Leila Azadbakht[2,8,9*]

Supplementary Table S1. PRISMA Checklist.

Section and Topic	Item #	Checklist item	Location where item is reported
**TITLE**			
Title	1	Identify the report as a systematic review.	1
**ABSTRACT**			
Abstract	2	See the PRISMA 2020 for Abstracts checklist.	2
**INTRODUCTION**			
Rationale	3	Describe the rationale for the review in the context of existing knowledge.	4
Objectives	4	Provide an explicit statement of the objective(s) or question(s) the review addresses.	4
**METHODS**			
Eligibility criteria	5	Specify the inclusion and exclusion criteria for the review and how studies were grouped for the syntheses.	5
Information sources	6	Specify all databases, registers, websites, organisations, reference lists and other sources searched or consulted to identify studies. Specify the date when each source was last searched or consulted.	4
Search strategy	7	Present the full search strategies for all databases, registers and websites, including any filters and limits used.	Supplement table 3
Selection process	8	Specify the methods used to decide whether a study met the inclusion criteria of the review, including how many reviewers screened each record and each report retrieved, whether they worked independently, and if applicable, details of automation tools used in the process.	5
Data collection process	9	Specify the methods used to collect data from reports, including how many reviewers collected data from each report, whether they worked independently, any processes for obtaining or confirming data from study investigators, and if applicable, details of automation tools used in the process.	6
Data items	10a	List and define all outcomes for which data were sought. Specify whether all results that were compatible with each outcome domain in each study were sought (e.g. for all measures, time points, analyses), and if not, the methods used to decide which results to collect.	6
	10b	List and define all other variables for which data were sought (e.g. participant and intervention characteristics, funding sources). Describe any assumptions made about any missing or unclear information.	6
Study risk of bias assessment	11	Specify the methods used to assess risk of bias in the included studies, including details of the tool(s) used, how many reviewers assessed each study and whether they worked independently, and if applicable, details of automation tools used in the process.	6
Effect measures	12	Specify for each outcome the effect measure(s) (e.g. risk ratio, mean difference) used in the synthesis or presentation of results.	6
Synthesis methods	13a	Describe the processes used to decide which studies were eligible for each synthesis (e.g. tabulating the study intervention characteristics and comparing against the planned groups for each synthesis (item #5)).	6
	13b	Describe any methods required to prepare the data for presentation or synthesis, such as handling of missing summary statistics, or data conversions.	7
	13c	Describe any methods used to tabulate or visually display results of individual studies and syntheses.	7

Section and Topic	Item #	Checklist item	Location where item is reported
	13d	Describe any methods used to synthesize results and provide a rationale for the choice(s). If meta-analysis was performed, describe the model(s), method(s) to identify the presence and extent of statistical heterogeneity, and software package(s) used.	7
	13e	Describe any methods used to explore possible causes of heterogeneity among study results (e.g. subgroup analysis, meta-regression).	7
	13f	Describe any sensitivity analyses conducted to assess robustness of the synthesized results.	7
Reporting bias assessment	14	Describe any methods used to assess risk of bias due to missing results in a synthesis (arising from reporting biases).	6
Certainty assessment	15	Describe any methods used to assess certainty (or confidence) in the body of evidence for an outcome.	6
**RESULTS**			
Study selection	16a	Describe the results of the search and selection process, from the number of records identified in the search to the number of studies included in the review, ideally using a flow diagram.	8
	16b	Cite studies that might appear to meet the inclusion criteria, but which were excluded, and explain why they were excluded.	8
Study characteristics	17	Cite each included study and present its characteristics.	9
Risk of bias in studies	18	Present assessments of risk of bias for each included study.	Supplemental Figure 10,11
Results of individual studies	19	For all outcomes, present, for each study: (a) summary statistics for each group (where appropriate) and (b) an effect estimate and its precision (e.g. confidence/credible interval), ideally using structured tables or plots.	Figure 2,3,4
Results of syntheses	20a	For each synthesis, briefly summarise the characteristics and risk of bias among contributing studies.	Supplemental Figure 10,11
	20b	Present results of all statistical syntheses conducted. If meta-analysis was done, present for each the summary estimate and its precision (e.g. confidence/credible interval) and measures of statistical heterogeneity. If comparing groups, describe the direction of the effect.	Figure 2,3,4,5
	20c	Present results of all investigations of possible causes of heterogeneity among study results.	11
	20d	Present results of all sensitivity analyses conducted to assess the robustness of the synthesized results.	12
Reporting biases	21	Present assessments of risk of bias due to missing results (arising from reporting biases) for each synthesis assessed.	9
Certainty of evidence	22	Present assessments of certainty (or confidence) in the body of evidence for each outcome assessed.	9
**DISCUSSION**			
Discussion	23a	Provide a general interpretation of the results in the context of other evidence.	13
	23b	Discuss any limitations of the evidence included in the review.	16
	23c	Discuss any limitations of the review processes used.	16

Section and Topic	Item #	Checklist item	Location where item is reported
	23d	Discuss implications of the results for practice, policy, and future research.	16
**OTHER INFORMATION**			
Registration and protocol	24a	Provide registration information for the review, including register name and registration number, or state that the review was not registered.	8
	24b	Indicate where the review protocol can be accessed, or state that a protocol was not prepared.	8
	24c	Describe and explain any amendments to information provided at registration or in the protocol.	8
Support	25	Describe sources of financial or non-financial support for the review, and the role of the funders or sponsors in the review.	17
Competing interests	26	Declare any competing interests of review authors.	16
Availability of data, code and other materials	27	Report which of the following are publicly available and where they can be found: template data collection forms; data extracted from included studies; data used for all analyses; analytic code; any other materials used in the review.	17

Supplementary Table S2. MOOSE Checklist.

	Reported on page	Comments
**Reporting of background should include**		
Problem definition	3-4	Introduction
Hypothesis statement	3-4	Introduction
Description of study outcomes	3-4	Introduction, Methods
Type of exposure or intervention used	3-4	Introduction
Type of study designs used	3-4	Methods
Study population	3-4	Introduction
**Reporting of search strategy should include**		
Qualifications of searchers (eg librarians and investigators)	5	Methods
Search strategy, including time period used in the synthesis and key words	5	Methods
Effort to include all available studies, including contact with authors	5	Methods
Databases and registries searched	5	Methods
Search software used, name and version, including special features used (eg explosion)	Did not use search software	Methods
Use of hand searching (eg reference lists of obtained articles)	5	
List of citations located and those excluded, including justification	Figure 1	
Method of addressing articles published in languages other than English	Did not include	
Method of handling abstracts and unpublished studies	Did not include	
Description of any contact with authors	Did not contact author	
**Reporting of methods should include**		
Description of relevance or appropriateness of studies assembled for assessing the hypothesis to be tested	4-5	Methods
Rationale for the selection and coding of data (eg sound clinical principles or convenience)	4-5	Methods
Documentation of how data were classified and coded (eg multiple raters, blinding and interrater reliability)	5	Methods
Assessment of confounding (eg comparability of cases and controls in studies where appropriate)	5	Methods

Assessment of study quality, including blinding of quality assessors, stratification or regression on possible predictors of study results	6, 7	Methods
Assessment of heterogeneity	7	Methods
Description of statistical methods (eg complete description of fixed or random effects models, justification of whether the chosen models account for predictors of study results, dose-response models, or cumulative meta-analysis) in sufficient detail to be replicated	7	Methods
Provision of appropriate tables and graphics	Figures	Figure 1
**Reporting of results should include**		
Graphic summarizing individual study estimates and overall estimate	Figures and tables	Figure 2, 3, 4
Table giving descriptive information for each study included	Tables	Table 1
Results of sensitivity testing (eg subgroup analysis)	11	Results, Figure 1-7
Indication of statistical uncertainty of findings	Figures and tables	Figure 2, 3, 4
**Reporting of discussion should include**		
Quantitative assessment of bias (eg publication bias)	9	Results, Supplemental Figure 10,11
Justification for exclusion (eg exclusion of non-English language citations)	Figure 1	
Assessment of quality of included studies	9	Results
**Reporting of conclusions should include**		
Consideration of alternative explanations for observed results	16	Discussion
Generalization of the conclusions (eg appropriate for the data presented and within the domain of the literature review)	16	Discussion
Guidelines for future research	16	Discussion
Disclosure of funding source	Acknowledgement	Acknowledgement

Supplemental Table S3. Search strategy

Database	Search period	Search terms
**PubMed**		("Caffeine"[MeSH Terms] OR "Coffee"[MeSH Terms] OR "Coffea"[MeSH Terms] OR "Caffein*" OR "Coffe*") AND ("abortion, spontaneous"[MeSH Terms] OR "stillbirth"[MeSH Terms] OR "abortion*" OR "miscarriage*" OR "stillbirth*" OR "still birth*" OR "fetal death*" OR "fetal loss*" OR "pregnancy loss*" OR misbirth*) AND (Prospective* OR retrospective* OR observational OR longitudinal OR cohort* OR "relative risk" OR "hazard ratio" OR "odds ratio" OR follow-up OR "follow up" OR population-based OR hr OR rr)
**Scopus**		(ALL("Caffein*" OR "Coffe*") AND ALL(abortion* OR miscarriage* OR stillbirth* OR "still birth*" OR "fetal death*" OR "fetal loss*" OR "pregnancy loss*" OR misbirth*) AND TITLE-ABS-KEY(Prospective* OR retrospective* OR observational OR longitudinal OR cohort* OR "relative risk" OR "hazard ratio" OR "odds ratio" OR follow-up OR "follow up" OR population-based OR hr OR rr))
**Web of science**		ALL=("Caffein*" OR "Coffe*") AND ALL=(abortion* OR miscarriage* OR stillbirth* OR "still birth*" OR "fetal death*" OR "fetal loss*" OR "pregnancy loss*" OR misbirth*) AND ALL=(Prospective* OR retrospective* OR observational OR longitudinal OR cohort* OR "relative risk" OR "hazard ratio" OR "odds ratio" OR follow-up OR "follow up" OR population-based OR hr OR rr)

Supplemental Table S4. GRADE risk of bias assessment

Certainty assessment							№ of patients		Effect		Certainty	Importance
**№ of studies**	**Study design**	**Risk of bias**	**Inconsistency**	**Indirectness**	**Imprecision**	**Other considerations**	**[intervention]**	**[comparison]**	**Relative (95% CI)**	**Absolute (95% CI)**		
**Coffee intake before pregnancy cohort**												
4	observational studies	very serious[a]	not serious	not serious	serious[b]	none		3872/24731 (15.7%)	**RR 1.21** (1.01 to 1.43)	**33 more per 1,000** (from 2 more to 67 more)	⊕◯◯◯ Very low	CRITICAL
**Coffee intake during pregnancy cohort**												
8	observational studies	very serious[c]	not serious[d]	not serious	serious	dose response gradient		11266/244310 (4.6%)	**RR 1.28** (1.04 to 1.57)	**13 more per 1,000** (from 2 more to 26 more)	⊕⊕◯◯ Low	CRITICAL
**Caffeine intake before pregnancy cohort**												
5	observational studies	very serious[e]	not serious	not serious	serious[f]	none		4144/29861 (13.9%)	**RR 1.14** (0.91 to 1.43)	**19 more per 1,000** (from 12 fewer to 60 more)	⊕◯◯◯ Very low	CRITICAL
**Caffeine intake during pregnancy cohort**												
13	observational studies	very serious[g]	serious[h]	not serious	not serious	publication bias strongly suspected dose response gradient[i]		6144/126926 (4.8%)	**RR 1.58** (1.23 to 2.01)	**28 more per 1,000** (from 11 more to 49 more)	⊕◯◯◯ Very low	CRITICAL

CI: confidence interval; **RR:** risk ratio

Explanations

a. Very serious risk of bias since most of studies were at high risk of bias. Downgraded.

b. Serious imprecision since the 95%CI include the null value and bounds of the 95%CI <0.9 and 1.10. Downgraded

c. Very serious risk of bias since most of studies were at high risk of bias. Downgraded.

d. Serious inconsistency since I2=74%, Phet=0.01. However, the subgroup of studies in cohort design indicated a similar positive association (RR: 1.28, 95%CI: 1.08, 1.51; I2=29%). Not downgraded.

e. Serious imprecision since the 95%CI include the null value and bounds of the 95%CI <0.9 and 1.10. Downgraded

f. Very serious risk of bias since most of studies were at high risk of bias. Downgraded.

g. Serious imprecision since the 95%CI include the null value and bounds of the 95%CI <0.9 and 1.10. Downgraded

h. Very serious risk of bias since most of studies were at high risk of bias. Downgraded.

i. Serious inconsistency since I2=88%, Phet<0.001. Downgraded.

j. Visual inspection of funnel plot and Egger's regression test (P<0.001) revealed a significant publication bias among included studies. Downgraded.

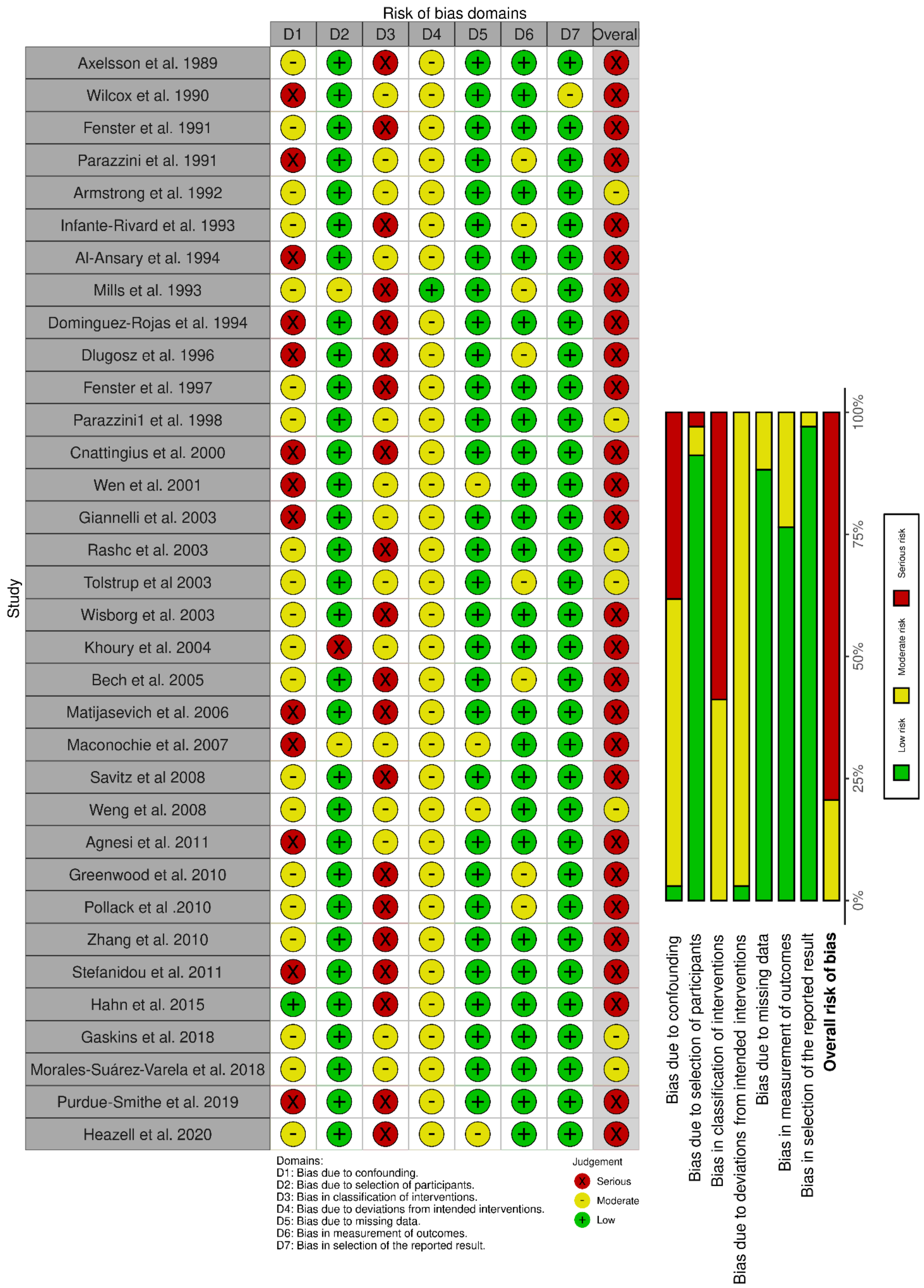

Supplemental Fig. 1 risk of bias assessment for each individual study comparison included in the meta-analysis. Colored bars represent the proportion of trial comparisons assessed as low (green), unclear (yellow) or high (red) risk of bias.

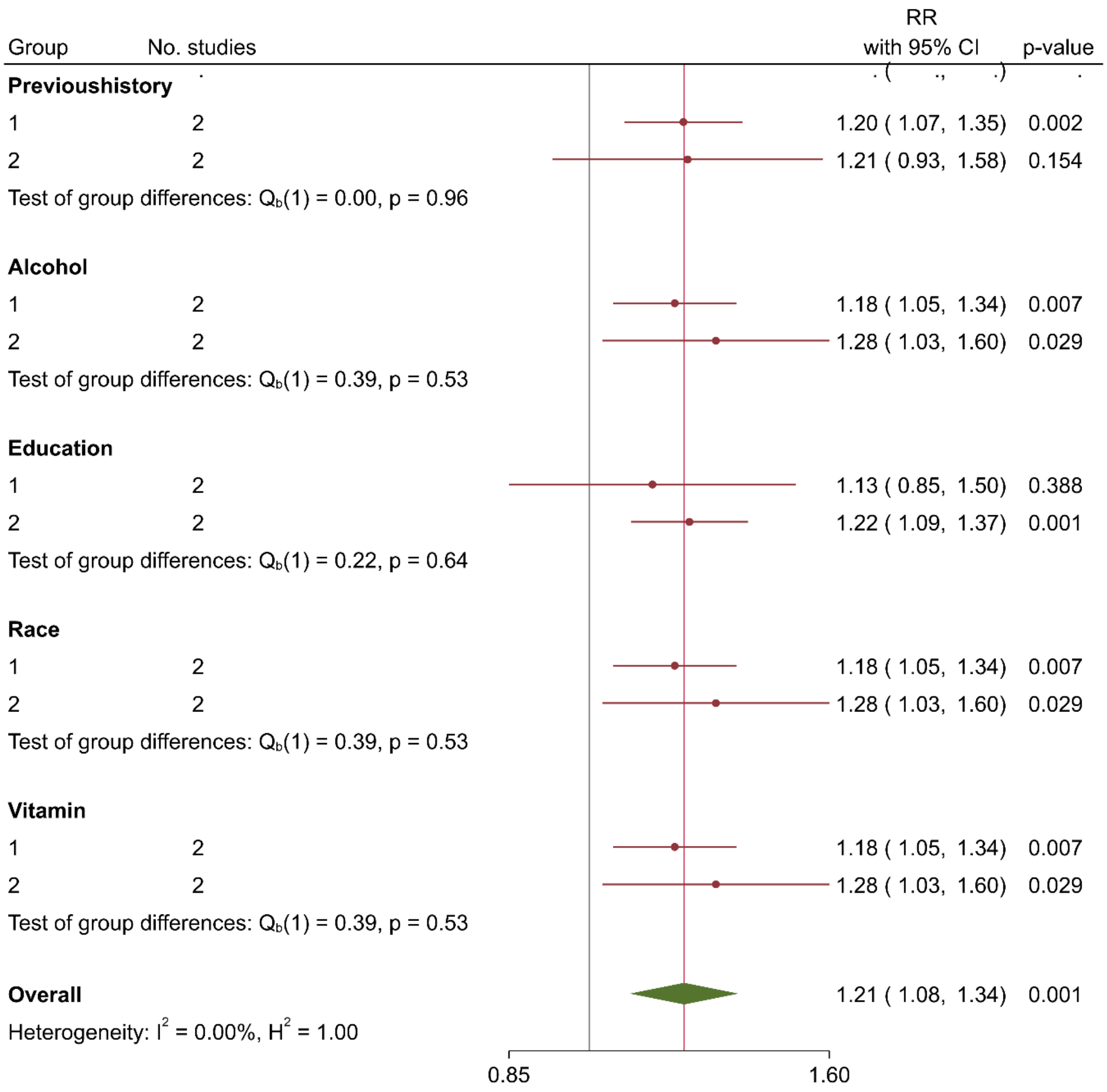

Supplemental Fig. 2. Subgroup analysis in the fix-effect model for the coffee intake before pregnancy

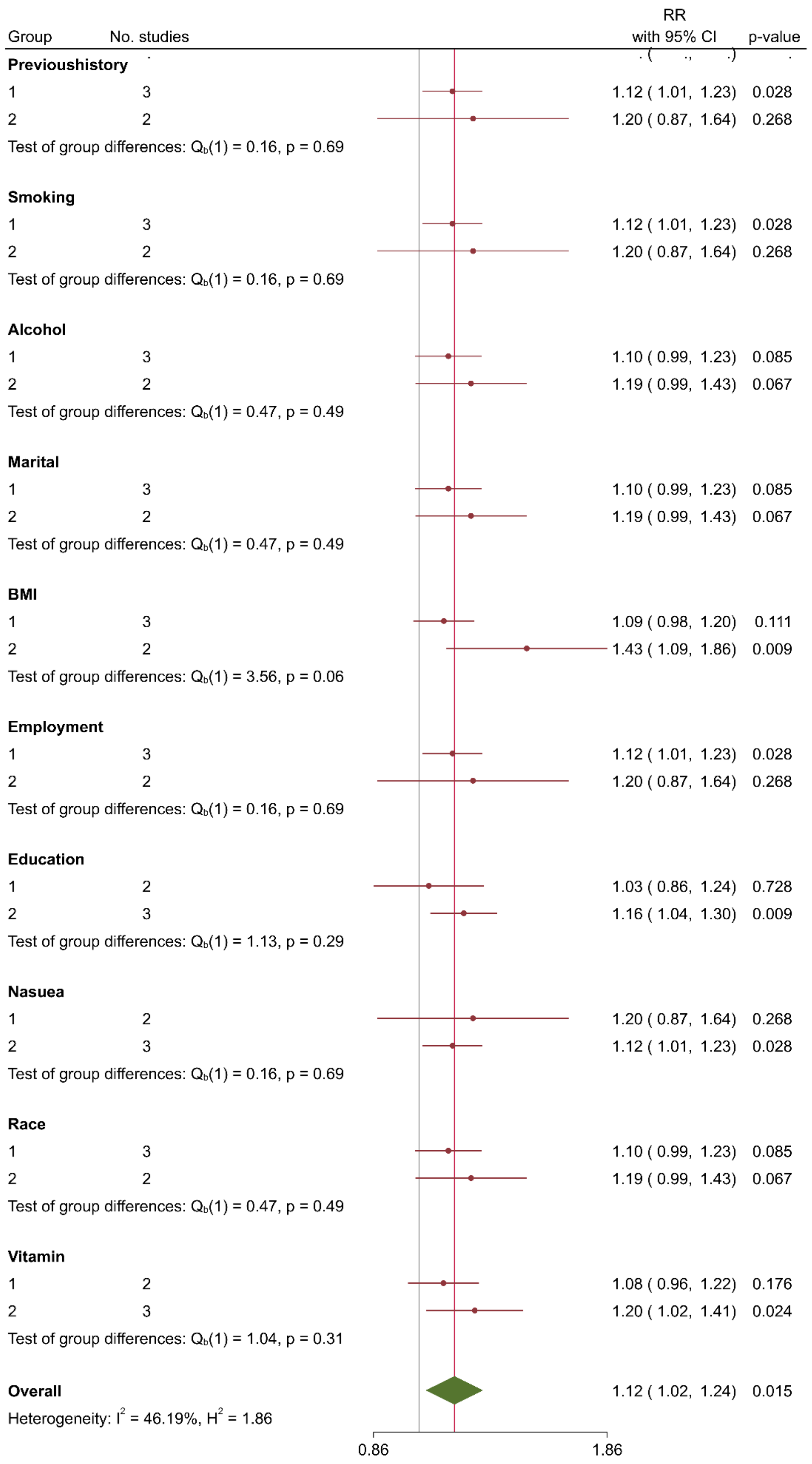

Supplemental Fig. 3. Subgroup analysis in the fix-effect model for the coffee intake during pregnancy in cohort studies

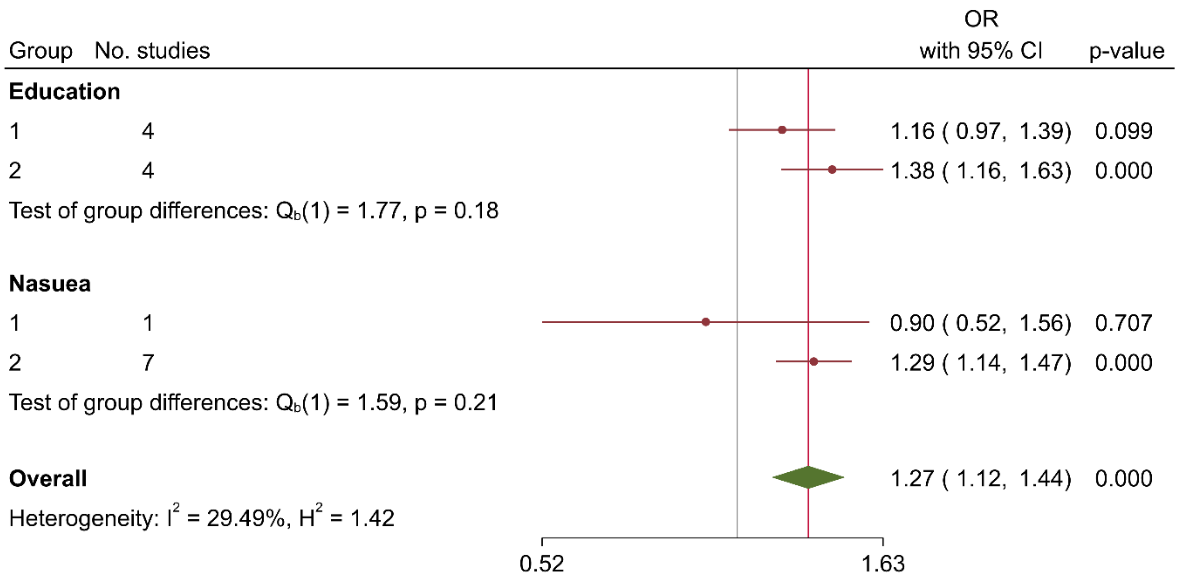

Supplemental Fig. 4. Subgroup analysis in the fix-effect model for the coffee intake during pregnancy in case-control

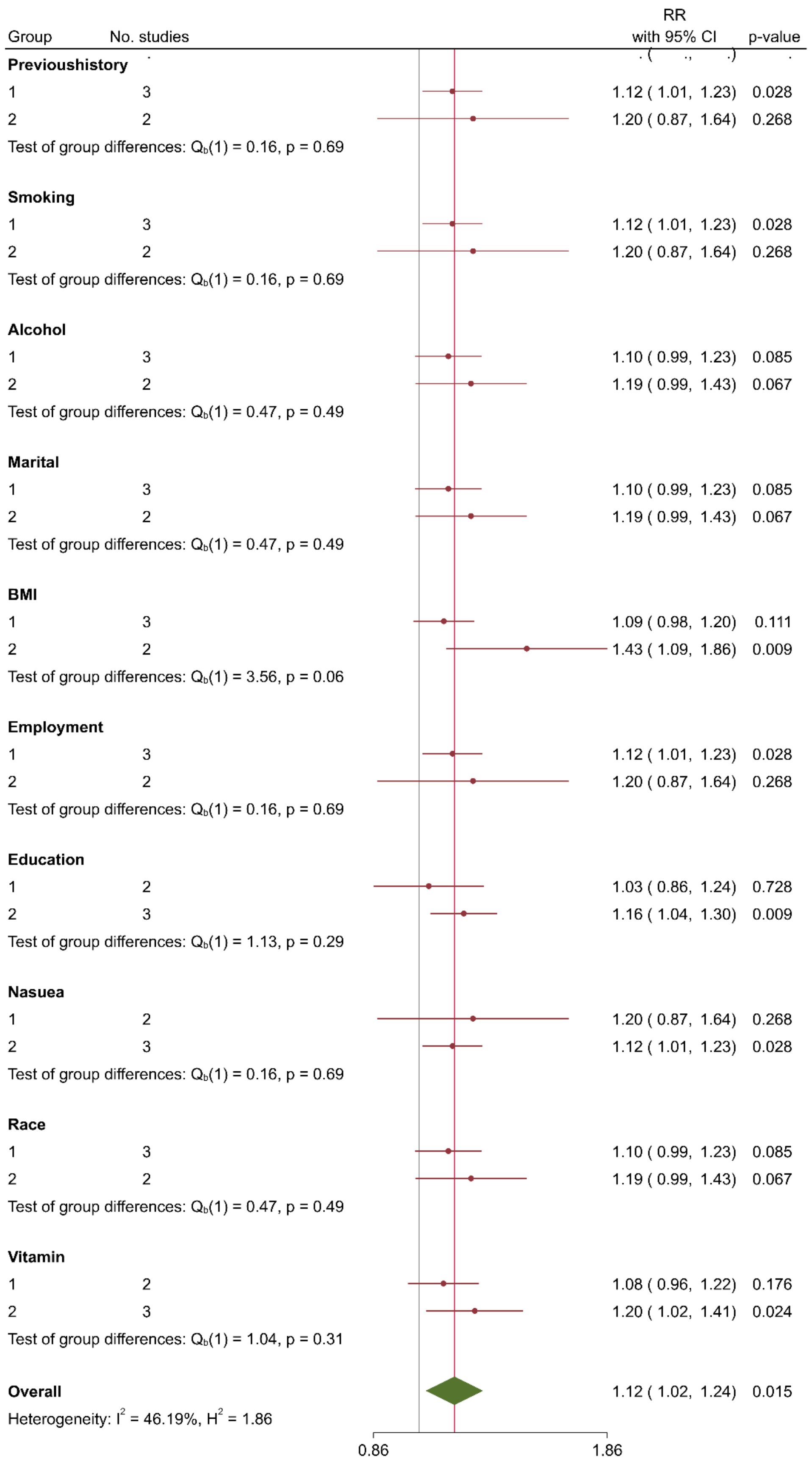

Supplemental Fig. 5. Subgroup analysis in the fix-effect model for the caffeine intake before pregnancy in cohort studies

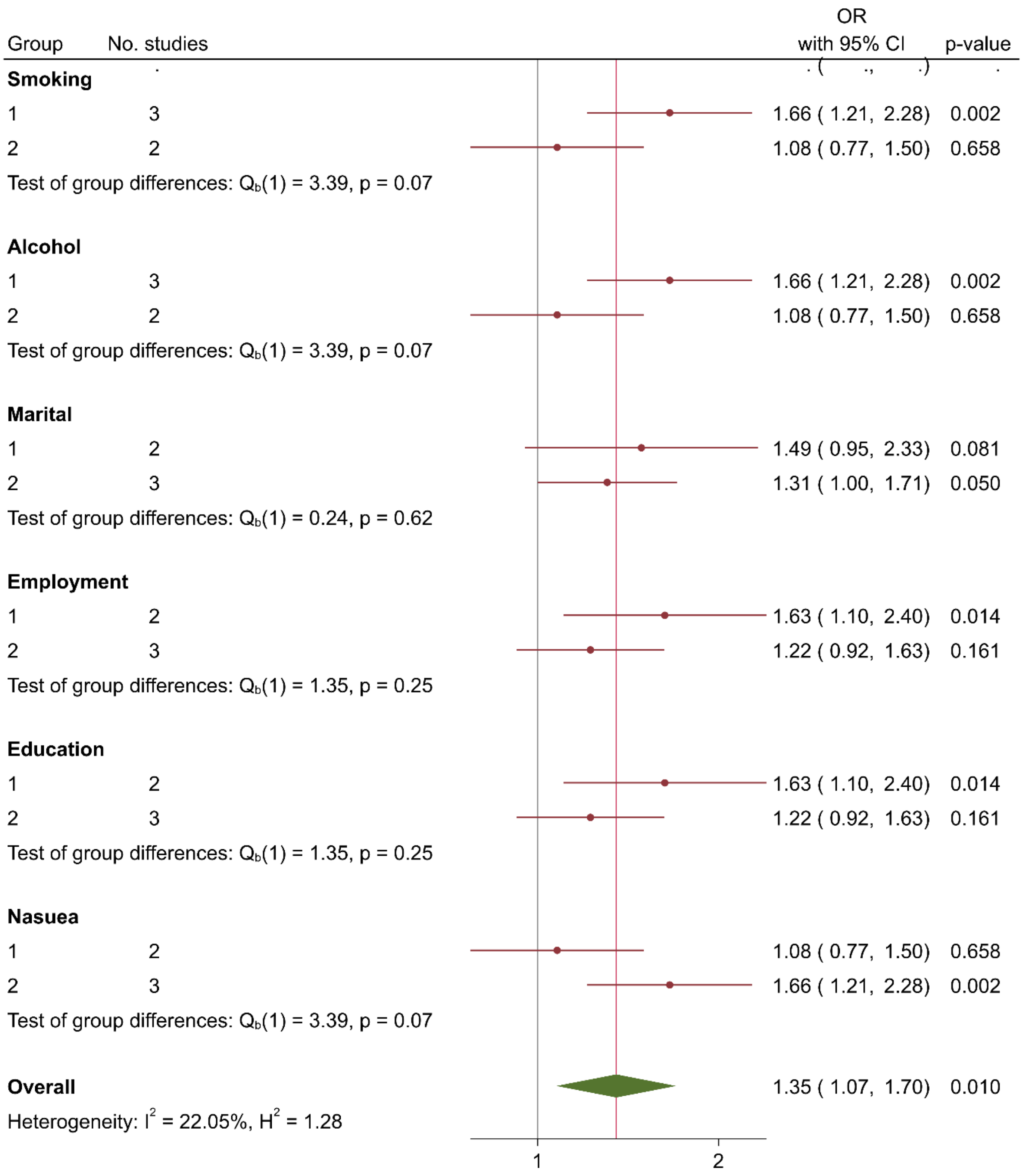

Supplemental Fig. 6. Subgroup analysis in the fix-effect model for the caffeine intake before pregnancy in case-control studies

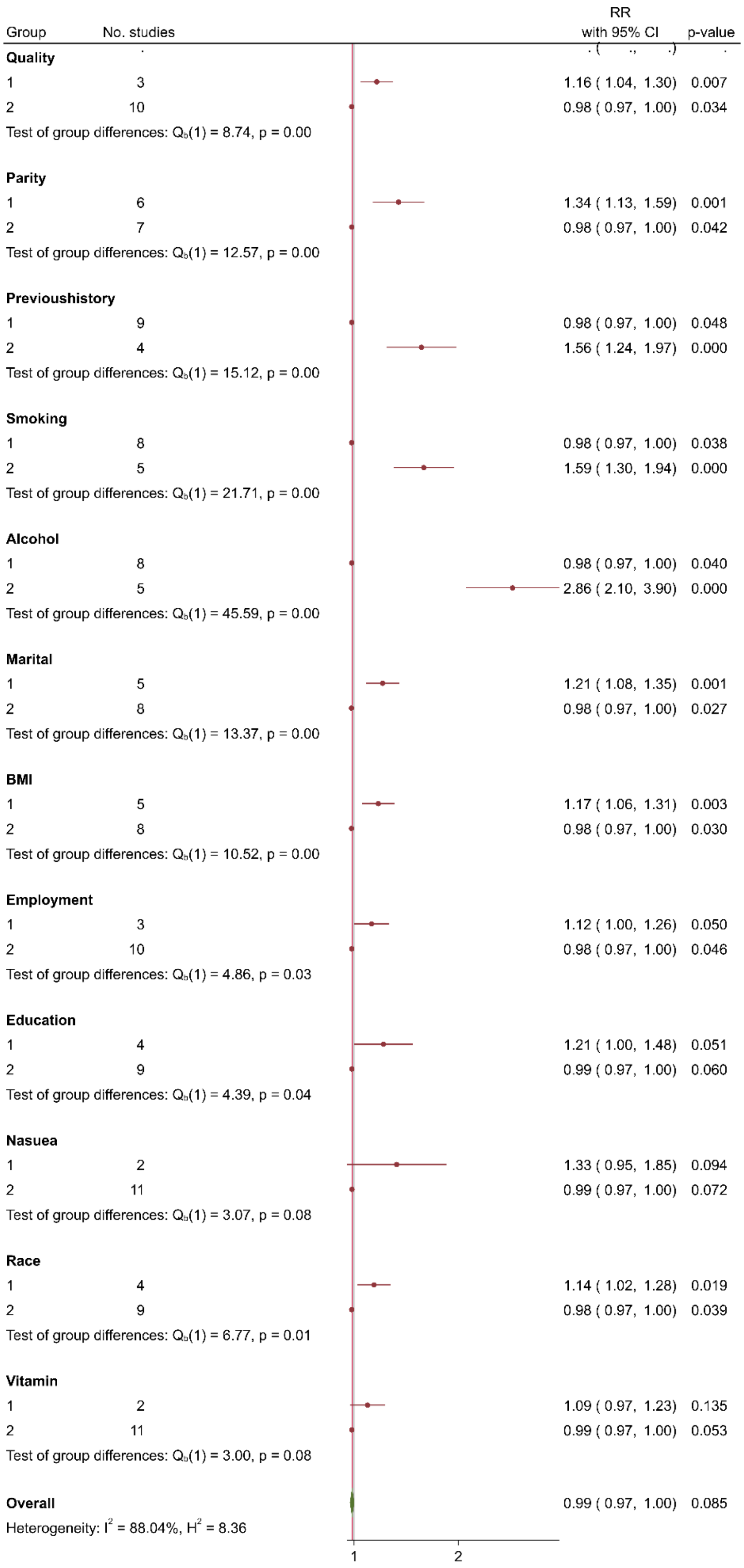

Supplemental Fig. 7. Subgroup analysis in the fix-effect model for the caffeine intake during pregnancy in cohort studies

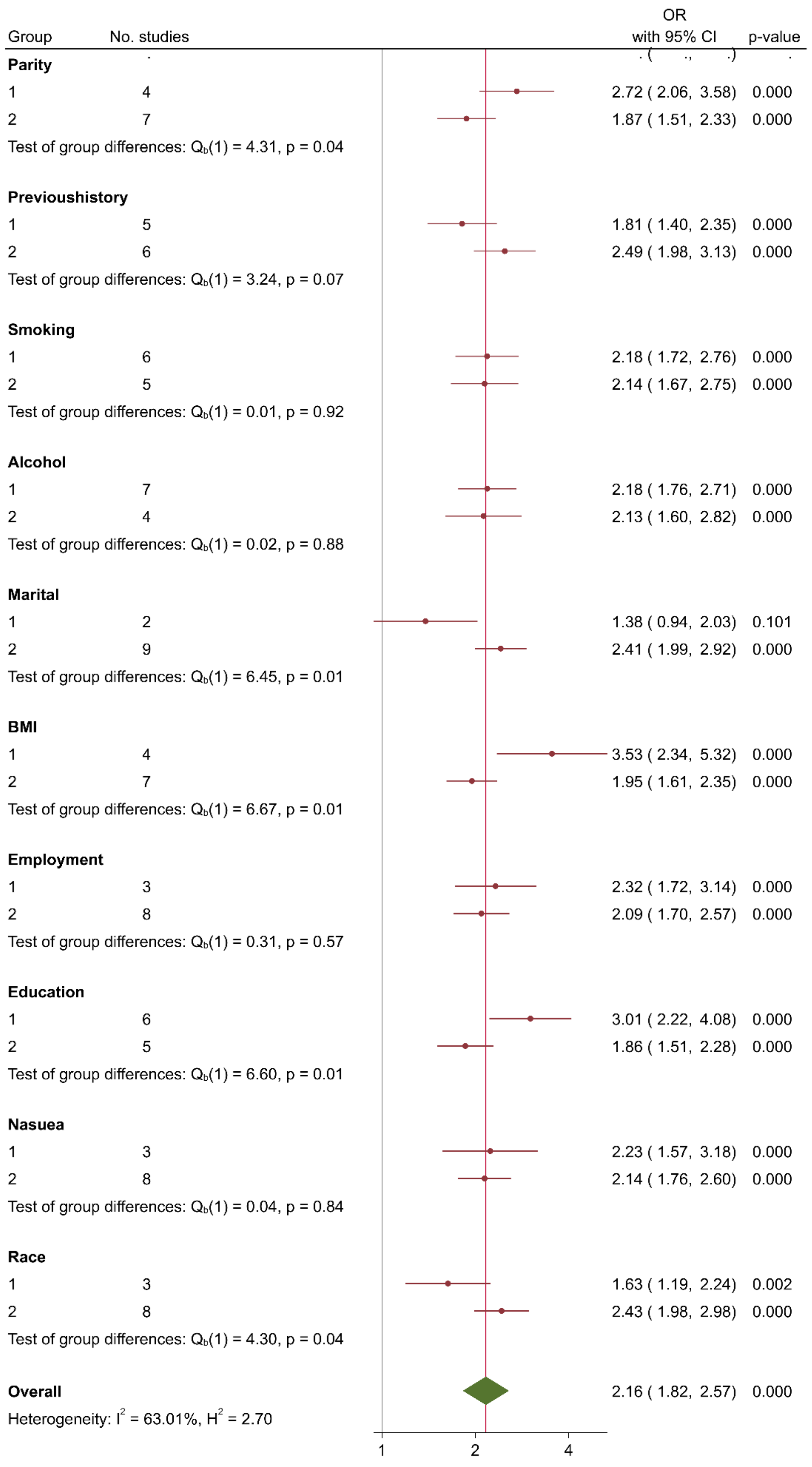

Supplemental Fig. 8. Subgroup analysis in the fix-effect model for the caffeine intake during pregnancy in case-control studies

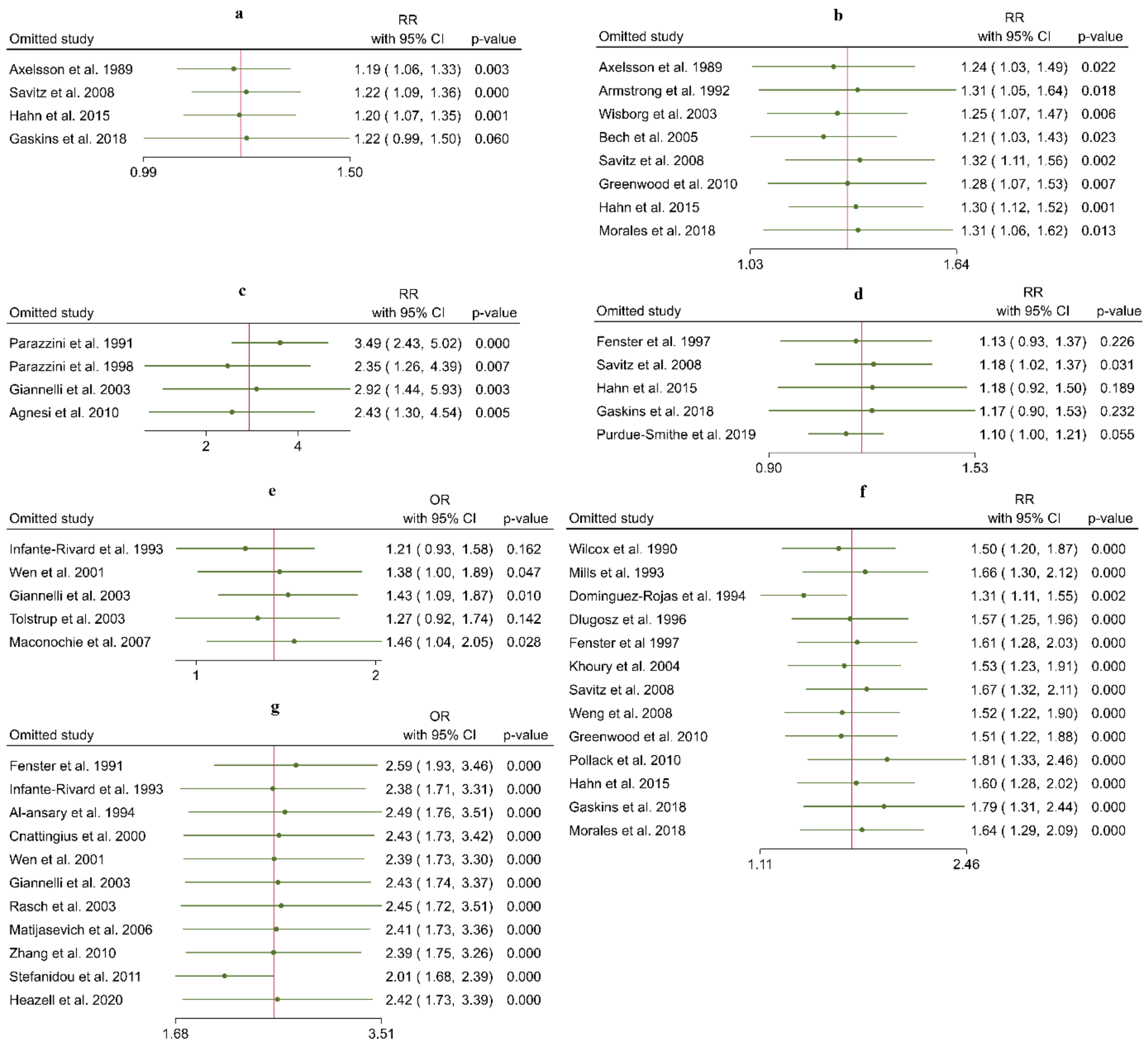

Supplemental Fig. 9. Sensitivity analysis of the systematic removal of each study comparison.

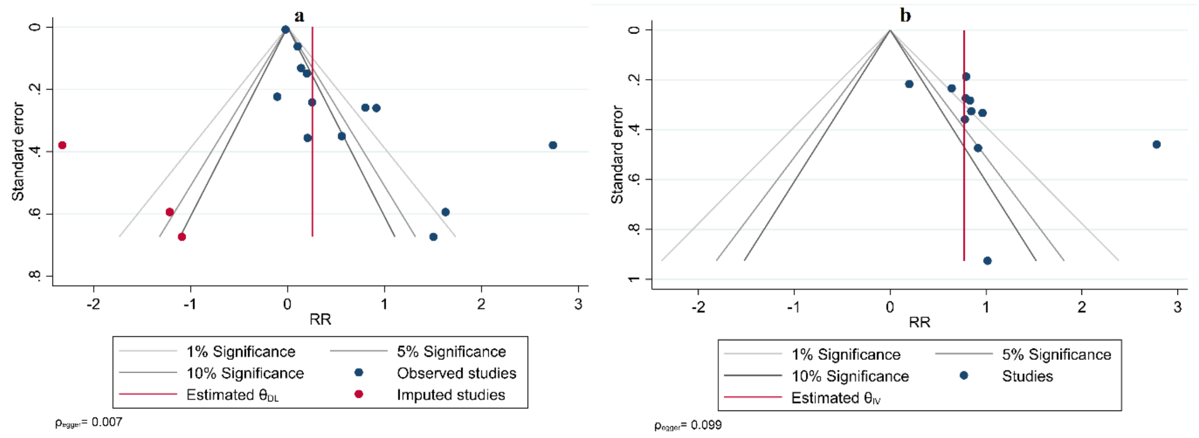

Supplemental fig. 10 Counter-enhanced funnel plot is a scatter-plot of each study comparison weighted mean difference on the x-axis with pseudo 95% confidence limits for caffeine intake during pregnancy with pregnancy loss in cohort and case-control studies. The vertical solid red line represents the pooled effect estimate and the dashed red lines represent the pseudo-95% confidence limits. The blue dots represent individual study comparisons. The contour regions define the regions for the test of significance of individual study effect size for a given p-value range >0.1, 0.5 to <0.1, 0.01 to <0.5, <0.01.

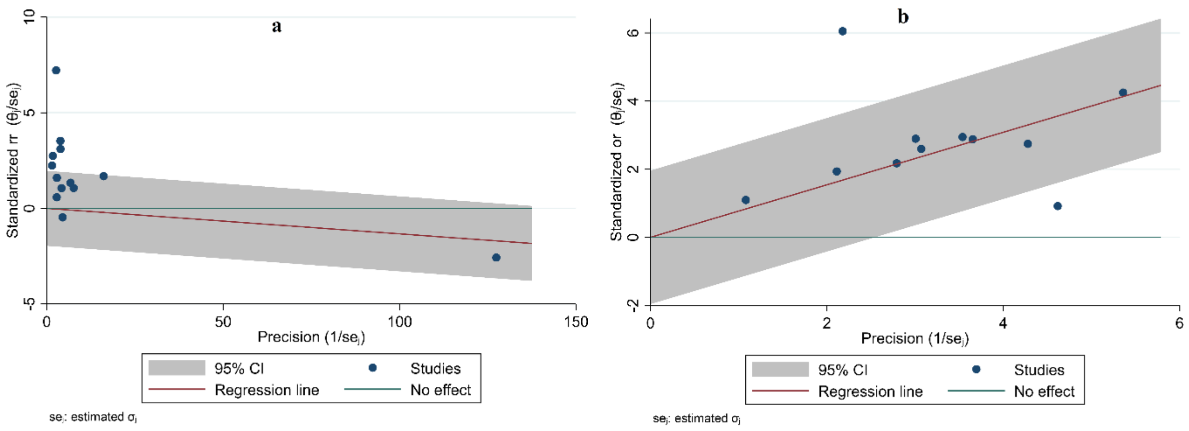

Supplemental fig. 11 Galbraith plot with pseudo 95% confidence limits for caffeine intake during pregnancy with pregnancy loss in cohort and case-control studies
